# Squamous Cell Carcinoma Originating From Mature Cystic Teratoma of the Ovary Diagnosed 10 Years After Initial Tumor Detection: A Case Report

**DOI:** 10.7759/cureus.76470

**Published:** 2024-12-27

**Authors:** Satoshi Ohira, Reika Kitano, Yuriko Yokoi, Fumiaki Kitamura, Akiko Hayashi

**Affiliations:** 1 Obstetrics and Gynecology, Marunouchi Hospital, Matsumoto, JPN

**Keywords:** malignant transformation, mature cystic teratoma, ovary, preoperative imaging, squamous cell carcinoma

## Abstract

Malignant transformation is a rare complication of ovarian mature cystic teratoma that occurs in 1-3% of cases. We herein report a case of squamous cell carcinoma originating from mature cystic teratoma of the ovary diagnosed 10 years after initial tumor detection. A 69-year-old woman presented to the Department of Internal Medicine with a seven-month history of abdominal fullness. Magnetic resonance imaging performed at another Department of Internal Medicine 10 years ago revealed a left ovarian teratoma measuring 8 cm; however, she refused referral to the Department of Gynecology. The current plane magnetic resonance imaging showed a large pelvic-abdominal cystic tumor measuring 17 cm, consisting of fat, fluid-fluid level, and a hair ball. Although the anterior wall of the cyst was slightly thick, the tumor had no solid projection into the cyst. She was referred to our Department of Gynecology. Contrast-enhanced abdominal computed tomography showed the lack of an enhanced solid component in the tumor. Laparotomy presented a large cystic tumor originating from the left ovary. A left salpingo-oophorectomy was performed without the intraperitoneal spillage of cyst fluid. Macroscopically, the left ovary was a unilocular cyst containing hair and sebaceous yellow fluid. A histological examination revealed that part of the thickened cyst wall was lined by squamous cell carcinoma *in situ*. Although the area of carcinoma *in situ* was dominant, nests of squamous cell carcinoma partially invaded the stroma. We diagnosed this tumor as squamous cell carcinoma originating from mature cystic teratoma of the left ovary. We speculate that the reason for the lack of enhancing solid components in the present case may be the abundance of squamous cell carcinoma *in situ*. The time required for mature cystic teratoma to become malignant remains unclear; therefore, the further accumulation of cases is needed to elucidate the mechanisms underlying the malignant transformation of mature cystic teratoma of the ovary.

## Introduction

Mature cystic teratoma is a recognized type of ovarian germ cell tumor [1]. Malignant transformation is a rare complication that occurs in 1-3% of cases [1]. Approximately 80% of the histological type of malignant transformation is squamous cell carcinoma (SCC) [2], and other malignancies include adenocarcinoma, sarcoma, carcinoid, thyroid carcinoma, and melanoma [3]. The malignant transformation of mature cystic teratoma is difficult to detect early, and the mechanisms underlying malignant transformation remain unclear [4]. We herein report a case of SCC originating from mature cystic teratoma of the ovary diagnosed 10 years after initial tumor detection. Moreover, the tumor of the present case lacked enhancing solid components in preoperative imaging findings.

## Case presentation

**Figure 1:**
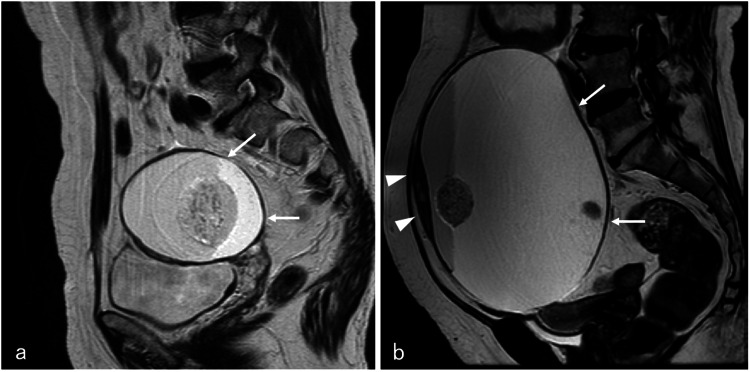
Ovarian tumor on MRI (a) MRI (T2-weighted imaging) performed 10 years ago revealed a left ovarian teratoma measuring 8 cm (arrows); (b) MRI (T2-weighted imaging) performed at our department showed a large cystic tumor measuring 17 cm, consisting of fat, fluid-fluid level, and a hair ball (arrows). The anterior wall of the cyst was slightly thick (arrowheads).

**Figure 2:**
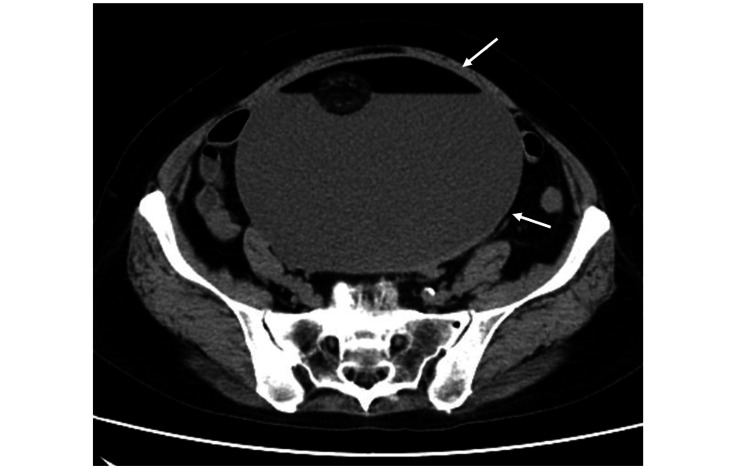
Ovarian tumor on CT Contrast-enhanced abdominal CT revealed the lack of an enhanced component in the tumor (arrows).

A 69-year-old postmenopausal woman (gravida 4, para 2, abortion 2) presented to the Department of Internal Medicine with a seven-month history of abdominal fullness. Although magnetic resonance imaging (MRI) performed at another Department of Internal Medicine 10 years ago revealed a left ovarian teratoma measuring 8 cm (Figure 1a), she refused referral to the Department of Gynecology. The current plane MRI showed a large pelvic-abdominal cystic tumor measuring 17 cm (Figure 1b); therefore, she was referred to our Department of Gynecology. The ultrasound revealed a large cystic mass with fluid-fluid level and a hair ball. Serum cancer antigen 125 and carbohydrate antigen 19-9 levels were elevated to 102.9 and 992.1 U/mL, respectively. Moreover, serum carcinoembryonic antigen and SCC antigen levels were high at 9.1 and 10.4 ng/mL, respectively. An MRI showed a large cystic tumor measuring 17 × 12 × 17 cm, consisting of fat, fluid-fluid level, and a hair ball. Although the anterior wall of the cyst was slightly thick, the tumor had no solid projection into the cyst (Figure 1b). We performed contrast-enhanced abdominal computed tomography (CT), which revealed the lack of an enhanced component in the tumor (Figure 2). Although serum tumor markers were above the normal ranges, we initially suspected benign mature cystic teratoma of the ovary based on imaging findings.

**Figure 3:**
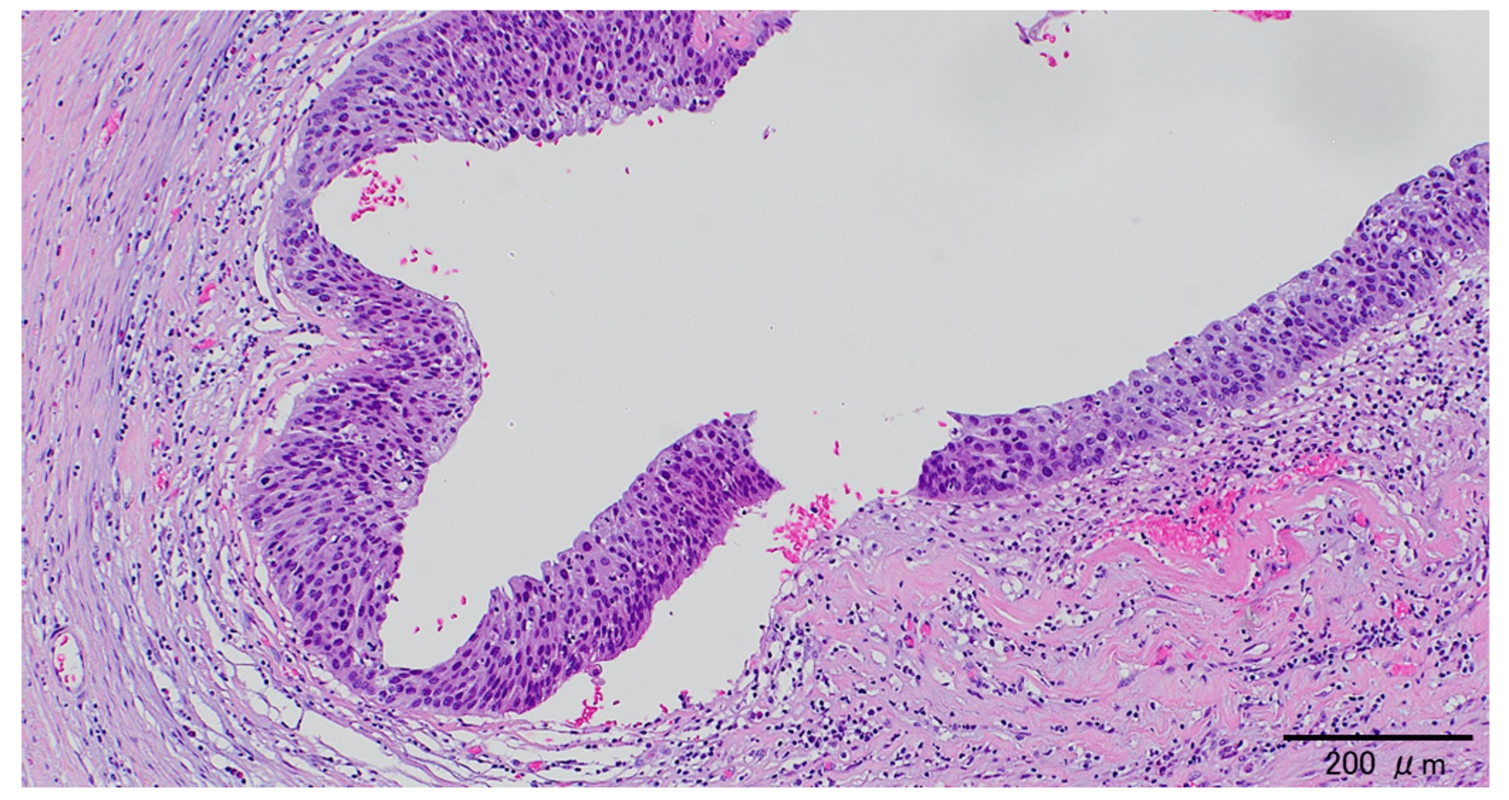
Squamous cell carcinoma in situ A histological examination showed that part of the thickened cyst wall was lined by a stratified squamous epithelium containing atypical cells throughout the entire epithelial thickness, consistent with SCC *in situ* (hematoxylin and eosin staining). SCC: squamous cell carcinoma

**Figure 4:**
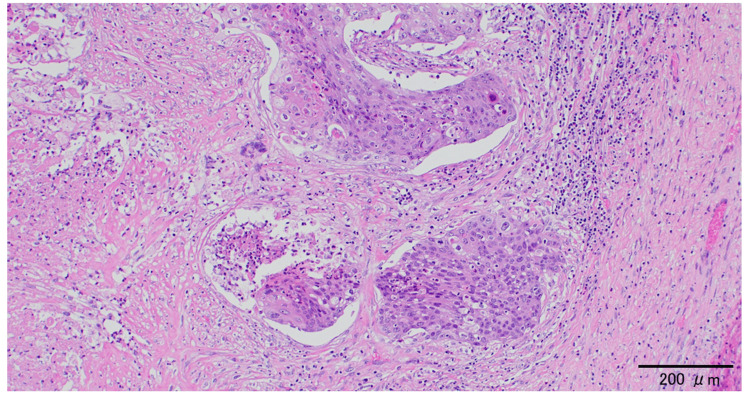
Invasive squamous cell carcinoma Nests of squamous cell carcinoma partially invaded the stroma (hematoxylin and eosin staining).

Laparotomy presented a large cystic tumor originating from the left ovary. The right ovary and uterus were unremarkable, and there was no peritoneal dissemination. Intraoperative ascitic cytology was negative. A frozen section analysis was unavailable, and a left salpingo-oophorectomy was performed without the intraperitoneal spillage of cyst fluid. Macroscopically, the surface of the left ovary was smooth, and there was an unilocular cyst containing hair and 1,260 mL of sebaceous yellow fluid. Although the cyst wall was thin, part of it was slightly thick. A histological examination revealed that the cyst wall was composed of hyalinized stroma, while part of the thickened cyst wall was lined by stratified squamous epithelium containing atypical cells throughout the entire epithelial thickness, consistent with SCC *in situ* (Figure 3). Although the area of carcinoma *in situ* was dominant, nests of SCC partially invaded the stroma (Figure 4). The left fallopian tube was unremarkable. We diagnosed the tumor as SCC arising from mature cystic teratoma of the left ovary. According to the 2008 International Federation of Gynecology and Obstetrics staging classification, the postoperative clinical diagnosis was stage IA ovarian cancer, pT1aNXMX.

We recommended restaging laparotomy to the patient, but she refused it. Although no metastatic lesions were found on chest and abdominal CT two months after surgery, the patient subsequently dropped out of the follow-up.

## Discussion

The mechanisms underlying the malignant transformation of mature cystic teratoma of the ovary have yet to be elucidated. Moreover, the time required for mature cystic teratoma to become malignant remains unclear. Similar to the present case, Jitsumori et al. reported a case of mature cystic teratoma that underwent malignant transformation 10 years after its initial detection [4]. Ovarian teratoma, in their case, increases in size from 6 to 21 cm in 10 years, and the postoperative pathological diagnosis was SCC arising from teratoma of stage IA. To the best of our knowledge, this is the second case report of mature cystic teratoma that underwent malignant transformation long after its initial detection.

A preoperative diagnosis of malignant transformation is important for patients with mature cystic teratoma of the ovary. Laparoscopic surgery is often conducted as treatment of mature cystic teratoma but has risks when malignant transformation is suspected [5]. Regarding imaging findings by CT and MRI, Park et al. reported that eight of 11 patients with malignant transformation had enhanced soft tissue components [6]. In another study using preoperative CT, Park et al. showed that five of eight cases of mature cystic teratoma with malignant transformation had enhanced nodular components [5]. Meanwhile, five of 15 cases of mature cystic teratoma without malignant transformation had nodular components, but none had enhanced components [5].

The tumor in the present case lacked enhanced solid components on preoperative CT. In previous studies on mature cystic teratoma cases, malignant transformation in some cases did not have enhanced solid components in preoperative imaging. Nevertheless, there have been a few well-documented case reports, such as the present case. We speculate that the reason for the lack of enhanced solid components in the present case may be the abundance of SCC *in situ*. Since invasive SCC was partial, the tumor had no solid projection into the cyst. We may have detected the early stage of progression from SCC *in situ* to stromal invasion.

## Conclusions

We herein reported a rare case of SCC arising from mature cystic teratoma of the ovary diagnosed 10 years after initial tumor detection. Ovarian teratoma in the present case increased in size from 8 cm to 17 cm in 10 years, and the tumor lacked enhanced solid components in preoperative imaging findings. Since the time required for mature cystic teratoma to become malignant remains unclear, the further accumulation of cases is needed to elucidate the mechanisms underlying the malignant transformation of mature cystic teratoma of the ovary.
